# Japanese Encephalitis Vaccine Generates Cross-Reactive Memory T Cell Responses to Zika Virus in Humans

**DOI:** 10.1155/2022/8379286

**Published:** 2022-11-19

**Authors:** Ran Wang, Meng Zhang, Linlin Zhang, Mengjia Liu, Chao Shan, Jing An, Zhengde Xie

**Affiliations:** ^1^Beijing Key Laboratory of Pediatric Respiratory Infection Diseases, Key Laboratory of Major Diseases in Children, Ministry of Education, National Clinical Research Center for Respiratory Diseases, Laboratory of Infection and Virology, Beijing Pediatric Research Institute, Beijing Children's Hospital, National Center for Children's Health, Capital Medical University, Beijing 100045, China; ^2^Research Unit of Critical Infection in Children, 2019RU016, Chinese Academy of Medical Sciences, Beijing, China; ^3^Department of Pediatric Rehabilitation, Beijing Boai Hospital, School of Rehabilitation Medicine, Capital Medical University, China Rehabilitation Research Center, Beijing 100068, China; ^4^State Key Laboratory of Virology, CAS Key Laboratory of Special Pathogens, Wuhan Institute of Virology, Center for Biosafety Mega-Science, Chinese Academy of Sciences, Wuhan 430071, Hubei, China; ^5^Department of Microbiology, School of Basic Medical Sciences, Capital Medical University, Beijing 100069, China; ^6^Center of Epilepsy, Beijing Institute for Brain Disorders, Beijing 100069, China

## Abstract

**Objective:**

Zika virus (ZIKV) and Japanese encephalitis virus (JEV) are mosquito-borne flaviviruses with sequence homology. ZIKV circulates in some regions where JEV also circulates, or where JE vaccination is used. Cross-immunity between flaviviruses exists, but the precise mechanisms remain unclear. We previously demonstrated that T cell immunity induced by the live-attenuated Japanese encephalitis (JE) SA14-14-2 vaccine conferred protective immunity against ZIKV infection in mice, which could even bypass antibody-dependent enhancement. However, the role of T cell immune, especially memory T cell subsets, in cross-reactive immune responses between JE vaccine and ZIKV in humans has not been reported.

**Methods:**

We examined central and effector memory CD4^+^ and CD8^+^ T cell (T_CM_ and T_EM_) responses (including degranulation, cytokines, and chemokines) in the presence of JEV and ZIKV, respectively, by using qualified peripheral blood mononuclear cell samples from 18 children who had recently received a two-dose course of JE vaccine SA14-14-2 as well as seven children without JE vaccination.

**Results:**

Cross-reactive CD8^+^ T_CM_ in response to ZIKV was characterized by secretion of IFN-*γ*, whereas CD8^+^ T_EM_ did not show significant upregulation of functional factors. In the presence of ZIKV, IFN-*γ* and TNF-*α* expression was upregulated by CD4^+^ T_EM_, and the expression signature of CD4^+^ T_CM_ is more cytotoxic potential.

**Conclusions:**

We profiled the cross-reactive memory T cell responses to ZIKV in JE vaccine recipients. These data will provide evidence for the mechanism of cross-reactive memory T cell immune responses between JEV and ZIKV and a more refined view of bivalent vaccine design strategy.

## 1. Introduction

Zika virus (ZIKV) and Japanese encephalitis virus (JEV) belong to the mosquito-borne flaviviruses [1]. Although ZIKV infection is mainly asymptomatic or mildly symptomatic, it can cause Guillain–Barre syndrome and other neurological syndromes in adults and serious fetal defects such as microcephaly [2, 3]. The epidemic caused by ZIKV has been declining since 2016, but ongoing transmission remains with the attendant risk of severe disease [4]. Reduced case numbers mean that there is still no licensed and available vaccine against ZIKV [5]. JEV is a flavivirus sharing some biological characteristics with ZIKV, which mostly causes asymptomatic infection or mild disease. However, JEV infection can also progress to acute Japanese encephalitis (JE), with a fatality rate of 20–30%, and 30–50% of recovered patients have sequelae [6]. Compared with the homology between DENV and ZIKV, JEV is more closely related to ZIKV [7, 8]. On average, JEV shares a 56.1% protein sequence identity with ZIKV. JEV is widely distributed in the Asia-Pacific region, in many countries in East, South, and Southeast Asia [9]. Among them, China was once the most affected country by JEV and one of the earliest countries to initiate JE-vaccination program [10].

Cross-reactive T cell responses induced by prior flavivirus exposure or vaccination to heterogeneous flaviviruses remains are widely reported between flaviviruses [11]. Unlike antibodies that can both prevent and enhance the subsequent infection with heterogeneous flaviviruses, the effect of cross-reactive T cell responses may be more inclined to protect against secondary infection [12–15]. With the ZIKV pandemic, the role of immunodominant protection of cross-reactive CD4^+^ and CD8^+^ T cells induced by primary DENV infection in secondary ZIKV infection has been described [13, 16, 17]. However, the degree of cross-reactivity and protective potential is influenced by factors such as the degree of homology, the sequence of infection, and the interval between primary and secondary infection. JEV has a much wider geographic range than DENV, previously for disease and now for vaccination, in China [18]. Since the implementation of the national Expanded Program on Immunization (EPI) in China in 2008, almost all Chinese people have been immunized against JE [19, 20], which prompted researchers to focus on the cross-reactive immune response between JEV and ZIKV. We and other groups have previously characterized the cross-reactive immune response between the two viruses. These studies suggest that the cross-protection is mainly conferred by the JEV-induced T cell response [15, 21]. These conclusions are mostly drawn from experiments in mice. ZIKV-specific cytotoxic CD8^+^ T cells can effectively suppress ZIKV infection [22]. In a model of CD8^+^ T cell adoptive transfer in mice, JE SA14-14-2-vaccination-induced CD8^+^ T cells can bypass or resist the ADE-mediated by cross-reactive antibodies, biasing the pathogenesis protection balance in global ZIKV infection in favor of protection [21]. The CD4^+^ T cells evoked by the JE-vaccination do not serve as the most dominant protective components but trigger Th1/Th2 cytokine generation through recognizing conserved epitopes [23]. Amplified cross-reactive clones boosted subsequent ZIKV vaccine responses, resulting in a higher degree of virus clearance [23]. When mapping the DENV-ZIKV cross-reactive CD4^+^ T cell response, it was observed that Th1-type cytokines played a more prominent role in inhibiting ZIKV replication [13].

The live-attenuated vaccine SA14-14-2 and the inactivated vaccine (JE-VC/IXIARO) and chimeric vaccine (ChimeriVax-JE) derived from SA14-14-2 strain are widely used and studied worldwide [24, 25]. Given the potential for T cells to mediate a cross-protective response, coupled with the fact that ZIKV is still circulating or potentially at risk of spreading [26], understanding the cross-reactive T cell response in children vaccinated with SA14-14-2 against ZIKV will be critical in assessing its potential to protect against ZIKV infection. Such data will also provide important clues for the development of a bivalent vaccine against JEV/ZIKV aimed at inducing a robust T cell response, with good safety profile (avoiding ADE responses) and an effective T cell response. Therefore, in this study, we used peripheral blood mononuclear cell (PBMC) samples collected from children who were vaccinated with two doses of JE vaccine SA14-14-2 to detect cross-reactive central memory (T_CM_) and effector memory (T_EM_) to ZIKV among CD4^+^ and CD8^+^ T cell memory T lymphocyte responses including cytokine secretion and degranulation upon ZIKV antigen stimulation, respectively [27–29].

## 2. Materials and Methods

### 2.1. Ethical Approval

Written informed consent in Chinese was obtained from all guardians of vaccinated children before enrollment, and the ethical approval was given by Beijing Children's Hospital, Capital Medical University (approval number: 2020-k-85). All procedures performed were in accordance with the Declaration of Helsinki. The study was explained in detail, and a section of the consent granted the investigators' permission for possible future use of the serum and PBMC samples.

### 2.2. Study Cohort

In total, we took peripheral venous blood samples and separated sera and PBMCs from 18 apparently healthy children (2 years old) who had previously received a prime and boost vaccination with live-attenuated JE SA14-14-2 vaccine for less than half a year from Jan through Feb 2022. Seven unvaccinated children's (6 months old) PBMCs were used as system controls. None of the subjects had visited an area where ZIKV was endemic and had no history of seeking medical attention for symptomatic ZIKV infection. The sex and age of study individuals are shown in Table S1. Before we analyzed the induction of JEV-specific and ZIKV cross-reactive CD4^+^ or CD8^+^ memory T cells among JE vaccinated individuals, hemogram parameters were analyzed in an automatic analyzer (Lifotronic Technology Co., Ltd.) within one hour after the blood samples were taken. There were no individuals with elevated C-reactive protein above the threshold. Seroconversion was confirmed by both enzyme-linkedimmune-sorbent assay (ELISA) and plaque reduction neutralization test (PRNT).

### 2.3. ELISA

The presence of JEV-specific IgG antibodies was measured by using an indirect ELISA kit (Shanghai B&C Biological Technology, China) according to the manufacturers' instructions, and it was previously described [20, 30]. Briefly, serum samples were diluted at 1 : 41 dilution with buffer that goes with the kit. The diluted test sera and control samples (100 *μ*L/well) were added to each well and incubated at 37°C for half an hour, followed by five washes. Then, 100 *μ*L of horseradish peroxidase-conjugated mouse antihuman IgG monoclonal antibody was added to each well at 37°C for half an hour. The initiation of the peroxidase reaction occurred after incubation at 37°C for 15 min in the dark. The reaction was then halted by the addition of 50 *μ*L of 2 M sulfuric acid per well. The result was represented as optical density, which was read at 450 nm using an ELISA plate reader (Thermo, USA). The cut-off value was calculated based on the manufacturers' instructions. The optical density of recipients' sera greater than the cut-off value was considered positive.

### 2.4. Cell and Lines Viruses

C6/36 cells and Vero cells were used for virus propagation and PRNT, respectively. Vero cells were cultured in the MEM medium containing 5% fetal bovine serum; C6/36 cells were cultured in the RPMI-1640 medium containing 10% fetal bovine serum. JEV (Beijing-1 strain) and ZIKV (SMGC_1 strain) were propagated in C6/36 cells and stored in a −80°C freezer. The virus was inactivated by UV irradiation for 1 h. Inactivated viral particles were harvested from the culture supernatant of C6/36 cells that had been infected by JEV or ZIKV, concentrated by 8% polyethylene glycol precipitation and then purified from clarified extracts by ultracentrifugation at 100,000 × *g* for 3 h at 4°C.

### 2.5. PRNT

The PRNT is considered as gold standard for detecting neutralizing antibodies (nAbs) against flaviviruses after vaccination or natural infection [20]. Seroconversion of nAbs is an indicator that flavivirus vaccine-induced immune protection has been successfully established. Heat inactivated sera were two-fold serially diluted from 1 : 10 to 1 : 160. The diluted serum was mixed with an equal volume of 100 plaque forming units (PFU) of JEV and incubated for 1 h at 37°C. The mixture was incubated with Vero cells for 1 h. Cells with removal of the inoculum were cultured under the MEM overlay medium and visualized by crystal violet staining. PRNT_50_ was defined as the reciprocal of the highest serum dilution that produced a 50% reduction in mean plaque number serum compared to control wells containing virus alone. With reference to the guideline, PRNT_50_ titers ≥1 : 10 are considered positive [31].

### 2.6. *Ex Vivo* Intracellular Staining (ICS)

Venous blood samples were collected in EDTA-K_2_-anticoagulated tubes. PBMCs were isolated by using lymphocyte separation medium density gradients (Dakewe Biotech Co., Ltd., China) [28]. ICS was performed as described previously [29, 32, 33]. Briefly, a total of 3 × 10^6^ freshly extracted PBMCs were divided into three equal parts with 1 × 10^6^ cells, and the two parts were stimulated with concentrated inactivated JEV or ZIKV particles at a final concentration of 1 × 10^5^ PFU/mL in a final volume of 500 *μ*L (MOI = 0.1) for 16 h at 37°C in the presence of 1 *μ*g/mL monoclonal antibodies CD28 (clone: CD28.2) and CD49d (clone: 9F10), GolgiPlug, monensin, and surface stained with BV605-anti-CD107a (clone: H4A3). Dead cells were labeled using Zombie NIR™ Fixable Viability Kit. Surface markers, including BV650-anti-CD3 (clone: SK7), BUV395-anti-CD4 (clone: SK3), BV421-anti-CD8 (clone: SK1), BUV737-anti-CD27 (clone: L128), and BV480-anti-CD45RO (clone: UCHL1) were stained. Cells were then washed, fixed with Cytofix/Cytoperm™ Fixation/Permeabilization Solution (BD Biosciences, USA), and stained with FITC-anti-IFN-*γ* (clone: 4S.B3), PE-anti-TNF-*α* (clone: MAb11), BV785-anti-IL-2 (clone: MQ1-17H12), and APC-anti-MIP-1*α* (clone: W16009B). The remaining part of PBMCs as negative controls without concentrated virus particles stimulation but combined with CD28 and CD49d were run for each sample. All regents were from BioLegend (USA) unless otherwise stated. All samples were acquired on a BD FACSymphony™ (BD Biosciences, USA) flow cytometer and analyzed using FlowJo version 10 software (TreeStar, USA). Cytokine responses were background subtracted individually before further analysis.

### 2.7. Statistical Analysis

Statistical analysis was performed with SPSS Statistics version 17.0 (SPSS Software Inc., USA), and the figures were made with GraphPad Prism version 6 (GraphPad Software Inc., USA). The Mann–Whitney *U* test or Kruskal–Wallis test (for multiple comparisons) were used to compare variables between two groups. The chi-square test was used to assess differences in the composition ratio of pluripotent T_PF_ between stimuli. Statistical significance was set at ^*∗*^*P* < 0.05, ^*∗∗*^*P* < 0.01, and ^*∗∗∗*^*P* < 0.001. All of the tests were two tailed.

## 3. Results

### 3.1. Complete Blood Count, IgG, and nAb Results

After preliminary testing of blood and isolated serum samples, complete blood count results of the 16 vaccinated children were all within the reference interval. Both IgG binding and nAb antibody measurement against JEV showed seroconversion, following two doses of SA14-14-2 vaccine among these children (Table 1).

### 3.2. Memory CD8^+^ T Cells

**Figure 1:**
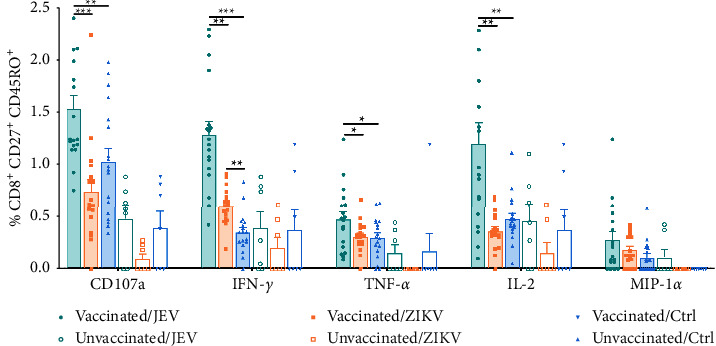
Multifunctional characterization of CD8^+^, CD27^+^ CD45RO^+^ central memory T cell (T_CM_) responses to JEV or ZIKV. CD8^+^ T_CM_ subpopulations were gated on cells expressing at least one of the five T cell functions analyzed, and the frequency of cells expressing any of the five cell functions was assessed. Vaccinated, *n* = 18; unvaccinated, *n* = 7. Results are expressed as mean ± SD. Differences between unmatched groups were compared using an unpaired *t*-test, the Mann–Whitney *U* test, or the Kruskal–Wallis rank-sum test with Dunn's post hoc test for multiple comparisons. ^*∗*^*P* < 0.05, ^*∗∗*^*P* < 0.01, and ^*∗∗∗*^*P* < 0.001.

**Figure 2:**
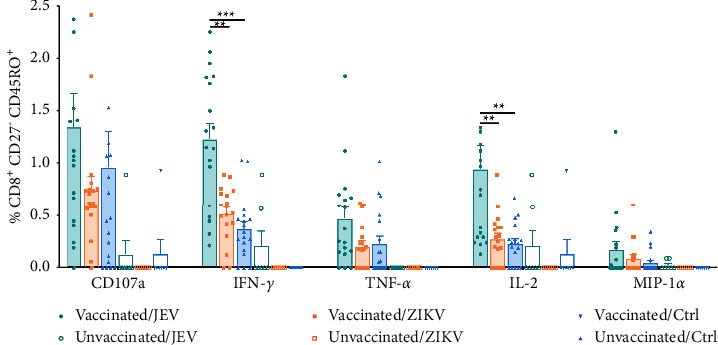
Multifunctional characterization of CD8^+^ CD27^−^ CD45RO^+^ effector memory T cell (T_EM_) responses to JEV or ZIKV. CD8^+^ T_EM_ subpopulations were gated on cells expressing at least one of the five T cell functions analyzed, and the frequency of cells expressing any of the five cell functions was assessed. Vaccinated, *n* = 18; unvaccinated, *n* = 7. Results are expressed as mean ± SD. Differences between unmatched groups were compared using an unpaired *t*-test, the Mann–Whitney *U* test, or the Kruskal–Wallis rank-sum test with Dunn's post hoc test for multiple comparisons. ^*∗∗*^*P* < 0.01; ^*∗∗∗*^*P* < 0.001.

To characterize and compare the functional response profiles of JEV-specific and cross-reactive CD8^+^ T cells to ZIKV in vaccinated children, we assessed the frequency, function, and the memory phenotype of memory CD8^+^ T cells by multicolor flow cytometry. The gating strategy is shown in Figure S1. For this analysis, we defined central memory CD8^+^ T cells as CD27^+^ CD45RO^+^ and effector memory CD8^+^ T cells as CD27^−^ CD45RO^+^, respectively [34]. We found that a large fraction of CD8^+^ T_CM_ cells in vaccinated children expressed CD107a (1.53% ± 0.54% vs. 1.03% ± 0.53%, *P* < 0.01) and IFN-*γ* (1.28% ± 0.53% vs. 0.35% ± 0.19%, *P* < 0.001) under JEV stimulation compared with controls, and higher expression of TNF-*α* (0.47% ± 0.30% vs. 0.30% ± 0.19%, *P* < 0.05) and IL-2 (1.20% ± 0.85% vs. 0.47% ± 0.23%, *P* < 0.01) were also detected, whereas MIP-1*α* did not show a significant increase (0.28% ± 0.32% vs. 0.11% ± 0.15%, *P* > 0.05, Figure 1). The frequencies of CD107a, IFN-*γ*, TNF-*α*, and IL-2 positive cells were higher after JEV stimulation than after ZIKV stimulation (*P* < 0.05). When pulsed with ZIKV, the responses of cross-reactive CD8^+^ T_CM_ in vaccinated children were significantly different from that of the control group in IFN-*γ* (0.60% ± 0.17% vs. 0.35% ± 0.19%, *P* < 0.01) but not in other indicators. T_EM_ cells are thought to exert antiviral effects directly upon restimulation with JEV. JEV-specific IFN-*γ* (1.23% ± 0.64% vs. 0.37% ± 0.30%, *P* < 0.001) positive cells were of dominant type responding to JEV, compared with those of the control group (Figure 2). Also, IL-2 (0.95% ± 0.95% vs. 0.23% ± 0.19%, *P* < 0.01) positive cells were detected with higher frequency after JEV stimulation. However, significantly elevated CD107a (1.35% ± 1.35% vs. 0.96% ± 1.45%, *P* > 0.05), TNF-*α* (0.47% ± 0.46% vs. 0.23% ± 0.32%, *P* > 0.05), and MIP-1*α* (0.17% ± 0.36% vs. 0.05% ± 0.10%, *P* > 0.05) were not detected. Responding cells expressing CD107a (0.75% ± 0.66% vs. 0.96% ± 1.45%, *P* > 0.05), IFN-*γ* (0.51% ± 0.27% vs. 0.37% ± 0.30%, *P* > 0.05), TNF-*α* (0.20% ± 0.22% vs. 0.23% ± 0.32%, *P* > 0.05), and IL-2 (0.24% ± 0.23% vs. 0.23% ± 0.19%, *P* > 0.05) were not significantly elevated after ZIKV stimulation. The level of IFN-*γ* in ZIKV cross-reactive T_EM_ was lower than in the JEV group (0.51% ± 0.27% vs. 0.37% ± 0.30%, *P* < 0.01). The commonality is that neither JEV nor ZIKV can stimulate CD8^+^ T_EM_ to secrete MIP-1*α*. These results suggest that only cross-reactive CD8^+^ T_CM_ is activated by ZIKV.

### 3.3. Memory CD4^+^ T Cells

**Figure 3:**
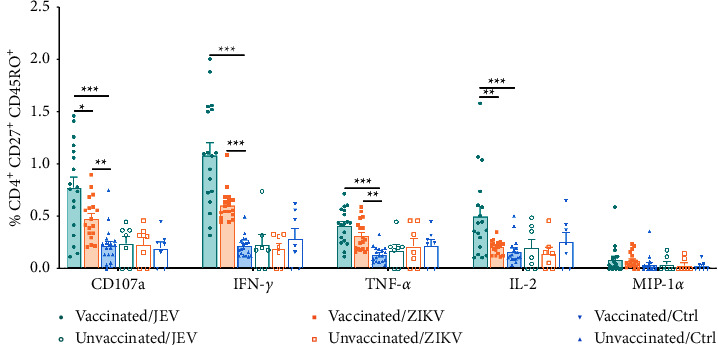
Multifunctional characterization of CD4^+^, CD27^+^, CD45RO^+^ central memory T cell (T_CM_) responses to JEV or ZIKV. CD4^+^ T_CM_ subpopulations were gated on cells expressing at least one of the five T cell functions analyzed, and the frequency of cells expressing any of the five cell functions was assessed. Vaccinated, *n* = 18; unvaccinated, *n* = 7. Results are expressed as mean ± SD. Differences between unmatched groups were compared using an unpaired *t*-test, the Mann–Whitney *U* test, or the Kruskal–Wallis rank-sum test with Dunn's post hoc test for multiple comparisons. ^*∗*^*P* < 0.05, ^*∗∗*^*P* < 0.01, and ^*∗∗∗*^*P* < 0.001.

**Figure 4:**
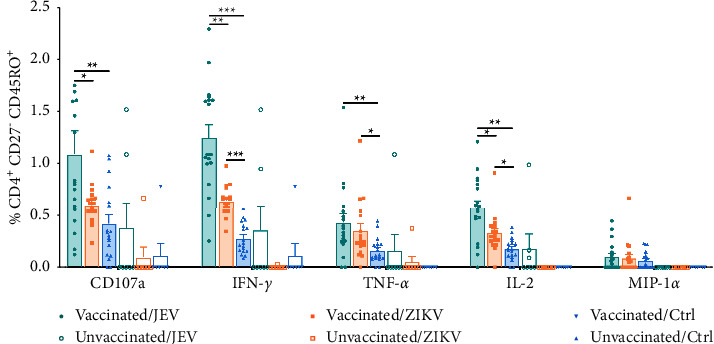
Multifunctional characterization of CD4^+^ CD27^−^ CD45RO^+^ effector memory T cell (T_EM_) responses to JEV or ZIKV. CD4^+^ T_EM_ subpopulations were gated on cells expressing at least one of the five T cell functions analyzed, and the frequency of cells expressing any of the five cell functions was assessed. Vaccinated, *n* = 18; unvaccinated, *n* = 7. Results are expressed as mean ± SD. Differences between unmatched groups were compared using an unpaired *t*-test, the Mann–Whitney *U* test, or the Kruskal–Wallis rank-sum test with Dunn's post hoc test for multiple comparisons. ^*∗*^*P* < 0.05, ^*∗∗*^*P* < 0.01, and ^*∗∗∗*^*P* < 0.001.

The role of CD4^+^ T cells in against flavivirus infection is also important. The simultaneous activation of memory CD4^+^ T cells and CD8^+^ T cells is the ideal cellular immune response. Therefore, we analyzed whether memory CD4^+^ T cell function could be cross-reactively evoked by ZIKV. Similar to CD8^+^ T_CM_, the proportion of IFN-*γ*^+^ and CD107a^+^ cells among the five functional subsets were higher in the presence of JEV (Figure 3). Compared with the control group, JEV antigen successfully induced higher levels of CD107a (0.78% ± 0.42% vs. 0.22% ± 0.18%, *P* < 0.001), IFN-*γ* (1.09% ± 0.49% vs. 0.22% ± 0.10%, *P* < 0.001), TNF-*α* (0.41% ± 0.17% vs. 0.14% ± 0.07%, *P* < 0.001), and IL-2 (0.51% ± 0.39% vs. 0.17% ± 0.11%, *P* < 0.001). Surprisingly, ZIKV stimulation appeared to trigger the expression of the CD107a, IFN-*γ*, and TNF-*α* and productions of CD107a (0.48% ± 0.20% vs. 0.22% ± 0.18%, *P* < 0.01), IFN-*γ* (0.61% ± 0.15% vs. 0.22% ± 0.10%, *P* < 0.01), and TNF-*α* (0.32% ± 0.14% vs. 0.14% ± 0.07%, *P* < 0.01). No significant increase in MIP-1*α* was detected in neither in JEV-specific nor in ZIKV cross-reactive CD4^+^ T_CM_ compared to the control group. It can be seen that CD4^+^ T_CM_ produced a broader cross-reactive cytokine profile after ZIKV stimulation than CD8^+^ T_CM_. CD4^+^ T_EM_ cells in the JEV group expressed high levels of CD107a (1.09% ± 0.94% vs. 0.41% ± 0.35%, *P* < 0.01), IFN-*γ* (1.25% ± 0.53% vs. 0.27% ± 0.14%, *P* < 0.001), TNF-*α* (0.42% ± 0.36% vs. 0.16% ± 0.12%, *P* < 0.01), and IL-2 (0.57% ± 0.31% vs. 0.17% ± 0.10%, *P* < 0.01) than those in the control group (Figure 4). As was the case for CD8^+^ T_EM_ cells, MIP-1*α* was also not expressed following stimulation with either virus. Notably, IFN-*γ* (0.64% ± 0.14% vs. 0.27% ± 0.14%, *P* < 0.001), TNF-*α* (0.35% ± 0.28% vs. 0.16% ± 0.12%, *P* < 0.05), and IL-2 (0.33% ± 0.18% vs. 0.17% ± 0.10%, *P* < 0.05) in CD4^+^ T_EM_ were detected after stimulation with ZIKV, when compared with control, although the frequencies of IFN-*γ* and IL-2 in the ZIKV group were still lower than that in the JEV group (*P* < 0.05). MIP-1*α* was not significantly increased upon stimulation with either JEV or ZIKV (*P* > 0.05).

## 4. Discussion

The role of T cell-mediated adaptive immune system in controlling viral infection should be of interest [35]. In addition to nAbs, T cells play an important role in host defense against viruses. As well as helping antibody responses, CD4^+^ T cells also aid in the initiation of cytotoxic T cells, the generation and maintenance of memory CD8^+^ T cells, as well as direct killing of target cells. CD8^+^ T cells can clear viruses from infected tissues by killing infected cells. For the optimal vaccine design, simultaneous activation of CD4^+^ and CD8^+^ T cells is an ideal strategy for vaccine-induced cellular immunity.

An earlier study reported that DENV-specific CD8^+^ and CD4^+^ T cells could produce IFN-*γ* upon flavivirus stimulation and lyse infected target cells [36]. Indeed, evidence accumulated from our group and other group's studies in mouse models suggests that T cells are actually protective against the flavivirus infection in both infection and vaccination settings, both in specific and cross-reactive responses [15, 32, 37, 38]. The immunization of immunodominant CD8^+^ T cell epitopes of DENV can improve viral clearance and protection during primary DENV infection [39]. CD8^+^ T cells can even confer protection from ADE-mediated infection with DENV and ZIKV in mice [21, 40, 41]. The protective effect of CD4^+^ T cells against flaviviruses has been clearly demonstrated in a mouse model [42]. Protective and long-lived immunity is closely related to the production of CD4^+^ T cells [43], which includes cytokine production, recruitment and activation of innate immune cells, enhancement of CD8^+^ T cell responses, promotion of immune memory, and direct cytotoxicity to infected cells [44]. Although some studies indicate that CD4^+^ T cells are not required for the control of primary DENV infection, their induction by epitope immunization nevertheless contributes to virus clearance and reduces tissue viral burden [38].

In humans, the exact role of JEV-induced T cells in preventing ZIKV infection and pathogenesis is unclear. We found that JEV-ZIKV cross-reactive T cells were detected in PBMC samples from children vaccinated with JEV, similar to our results in mice, and reported that these cells responded after restimulation *in vitro.* JEV-ZIKV cross-reactive CD8^+^ T_CM_ is only IFN-*γ*-producing upon ZIKV stimulation, but this cytokine appears to be critical in cross-protection in the mouse model. ZIKV cross-reactive CD8^+^ T_EM_ did not have detectable potential for cytotoxic and chemotactic activity. Here, in terms of CD4 memory T cells, we showed that among those who received two doses of SA14-14-2 vaccine, peripheral CD4^+^ T_CM_ and T_EM_ cells were characterized by the expression of three markers following ZIKV stimulation. Normally, T_CM_ are highly sensitive to antigenic stimulation, while the dependence on costimulatory signals is reduced. After homing to the T cell area of secondary lymphoid organs, T_CM_ cells present reactive memory and proliferate rapidly. They have almost no effector function but can proliferate stably and differentiate into effector T cells in the presence of antigen [45]. T_CM_ cells mainly produce IL-2, and a small amount of IFN-*γ* and perforin through T cell receptor signaling [46]. In this study, we observed a similar polyfunctional feature in cross-reactive CD4^+^ T_CM_ to that in the JEV group itself, with a high level of CD107a, IFN-*γ*, and TNF-*α*, indicating an important role for CD4^+^ T_CM_ in cross-reactive T cell responses. Cross-reactive CD4^+^ T_EM_ mainly expresses the markers IFN-*γ*, TNF-*α*, and IL-2. Studies have shown that immunity generated by flaviviruses sharing the CD4^+^ T cell epitope promotes protection during subsequent heterologous infection [12], which is speculated to be mediated by the NS3 protein [47, 48].

T cells express two or more of the above five markers, namely, polyfunctional T cells (T_PF_). We measured the coexpression of more than two markers; however, we found that the frequency of induction of ZIKV cross-reactive T_PF_ by JEV-vaccination was low. We detected two or more cytokine repertoires only in CD4^+^ T_CM_ and T_EM_ but not in CD8^+^ T_CM_ and T_EM_. In the CD4^+^ T_CM_ of JEV-vaccinated individuals, the frequency of the IFN^+^ TNF^+^ population was 0.02%, 0.01%, and 0.01% in the JEV-specific, ZIKV cross-reactive, and unstimulated groups, respectively, without differences across groups; in CD4^+^ T_EM_, the frequency of IFN^+^ TNF^+^ population was 0.04%, 0.01%, and 0% in these three groups, respectively.

It should be noted that existing anti-JEV antibody tests cannot completely rule out isolated ZIKV infection, as the available kits do have partial cross-reactivity to ZIKV in specificity, albeit at a very low level. In addition, the cases of latent infection with ZIKV have been detected in the population of Guangxi Province, a border province in southern China [26], bringing some uncertainty to the immune background of the study individuals in this study. However, we took into account the following three points: (1) the existing reported local cases of ZIKV infection were in border provinces in southern China but not yet prevalent in northern China, and these subjects did not travel outside of China in those ZIKV endemic areas; (2) they did not travel to the Chinese provinces (Yunnan and Guizhou) where ZIKV was detected in wild mosquitoes but no domestic ZIKV cases were reported; and (3) theoretically, it is unlikely that the level of T cell immune response caused by primary infection with ZIKV is lower than that of the cross-reaction elicited by JEV vaccination. Moreover, the COVID-19 pandemic poses considerable difficulties in the availability of larger sample sizes; thus, larger sample sizes would be beneficial to the firmness of the aforementioned conclusions.

Two issues to be considered in future investigations are explained. (1) To ensure that immune responses restricted by different HLA alleles and different species of JEV vaccines are adequately represented in JEV-ZIKV cross-reactive T cell response studies, the subjects of this study were all individuals vaccinated with the live-attenuated SA14-14-2 vaccine, but did not include individuals vaccinated with inactivated vaccines or recombinant chimeric vaccines, whose immune characteristics were different [49]. (2) The breadth of the immunodominant T cell epitope repertoire needs to be investigated, which has implications for the vaccine design, cross-reactivity, and immune escape by cross-reactive immune response. T cell epitopes of flaviviruses are generally conserved [43], and there are very few instances of T cell epitopes causing acute infections in viral escape (such as those caused by flaviviruses). In contrast, viruses that drive the progression of chronic viral infection evade T cell epitope recognition, which is due to a fundamental difference in selection pressure [50]. Given the importance of T cells in cross-reactive immune responses, boosting T cell responses to improve vaccine efficacy is desirable. This can be achieved by generating broad flavivirus cross-reactive T cell responses by sequential immunization against flaviviruses that share T cell epitopes.

## 5. Conclusions

In conclusion, we enrich our current understanding of how T cells induced in JEV-vaccinated children cross-react with ZIKV through experiments, and we put forward that the role of JEV-specific and ZIKV cross-reactive T cells in the infection control may be the strategy for the development of bivalent vaccines that induce dual protection with safety and efficacy. Further expansion of these findings will significantly improve our understanding of T cell function and highlight the potential clinical benefit of incorporating JEV-ZIKV cross-reactive T cell epitopes into experimental vaccine formulations to improve cellular immune responses.

## Figures and Tables

**Table 1:** Results of complete blood count and JEV antibody testing/titration of subjects in this study.

No.	Parameter											
	CRP (mg/L)	WBC count (×10^9^/L)	RBC count (×10^12^/L)	Hgb (g/L)	Plt count (×10^9^/L)	NEUT count (×10^9^/L)	LYMPH count (×10^9^/L)	MONO count (×10^9^/L)	EO count (×10^9^/L)	BASO count (×10^9^/L)	JEV IgG	JEV nAb
Reference interval (2 y–<6 y)	<8	4.4–11.9	4.0–5.5	112–149	188–472	1.2–7	1.8–6.3	0.12–0.93	0–0.68	0–0.1		
VAC 1	<8	5.94	4.21	120	274	1.49	3.92	0.38	0.13	0.02	+	1 : 80
VAC 2	<8	7.13	4.13	116	372	3.78	2.64	0.53	0.13	0.05	+	1 : 160
VAC 3	<8	9.84	4.46	119	348	6.93	2.41	0.49	0	0.01	+	1 : 20
VAC 4	<8	7.97	4.57	126	351	2.52	4.79	0.57	0.06	0.03	+	>1 : 160
VAC 5	<8	5.62	4.3	122	312	1.68	3.33	0.38	0.17	0.06	+	1 : 80
VAC 6	<8	6.87	4.7	126	400	1.62	4.54	0.55	0.13	0.03	+	1 : 40
VAC 7	<8	6.53	4.38	123	253	1.48	4.21	0.73	0.1	0.01	+	1 : 80
VAC 8	<8	5.29	4.15	120	275	1.64	3.1	0.38	0.14	0.03	+	1 : 80
VAC 9	<8	6.65	4.5	131	338	2.09	3.67	0.74	0.12	0.03	+	1 : 80
VAC 10	<8	8.53	4.9	129	371	3.63	4.32	0.43	0.11	0.04	+	1 : 80
VAC 11	<8	7.87	4.97	134	359	3.15	3.99	0.55	0.15	0.03	+	1 : 160
VAC 12	<8	5.39	4.67	123	226	2.78	2.2	0.33	0.06	0.02	+	1 : 40
VAC 13	<8	6.08	4.9	129	304	1.51	3.77	0.47	0.3	0.03	+	1 : 80
VAC 14	<8	7.09	4.14	113	361	3.24	3.09	0.55	0.18	0.03	+	>1 : 160
VAC 15	<8	8.49	5.01	141	281	2.14	5.73	0.42	0.16	0.04	+	1 : 20
VAC 16	<8	8.46	4.41	116	326	3.66	4.25	0.53	0.01	0.01	+	1 : 80
VAC 17	<8	8.6	4.85	130	271	1.77	6.22	0.51	0.06	0.04	+	1 : 160
VAC 18	<8	10.88	5.05	134	313	5.36	5.6	0.66	0.17	0.09	+	>1 : 160
Mean ± standard	—	7.4 ± 1.55	4.57 ± 0.32	125.11 ± 7.36	324.17 ± 60.26	2.8 ± 1.49	3.99 ± 1.12	0.51 ± 0.12	0.12 ± 0.07	0.03 ± 0.02		

Reference interval (28 d–< 6 m)	<8	4.3–14.2	3.3–5.2	97–183	183–614	0.6–7.5	2.4–9.5	0.15–1.56	0.07–1.02	0–0.1		
UNV 1	<8	9.87	3.54	99	292	4.79	3.9	1.02	1.02	0.07	−	<1 : 10
UNV 2	<8	8.62	4.65	138	257	6.92	3.97	0.72	0.08	0.01	−	<1 : 10
UNV 3	<8	11.5	5.1	169	377	5.62	5.81	1.23	0.38	0.03	−	<1 : 10
UNV 4	<8	9.47	3.65	125	405	2.6	4	1.08	0.77	0.02	−	<1 : 10
UNV 5	<8	10.95	4.92	172	302	4.7	3.96	1.01	0.36	0.04	−	<1 : 10
UNV 6	<8	6.4	3.57	109	258	0.67	3.86	0.6	0.26	0.01	−	<1 : 10
UNV 7	<8	5.87	3.88	112	247	1.50	3.61	0.56	0.08	0.02	−	<1 : 10
Mean ± standard	—	8.95 ± 2.15	4.19 ± 0.68	132 ± 29.07	305.43 ± 62.23	3.83 ± 2.29	4.16 ± 0.74	0.89 ± 0.26	0.42 ± 0.35	0.03 ± 0.02		

BASO: basophil, CRP: C-reaction protein, EO: eosinophil, Hgb: hemoglobin, LYMPH: lymphocyte, MONO: monocyte, NEUT: neutrophil, Plt: platelet, RBC: red blood cell, UNV: unvaccinated individuals, VAC: vaccinated individuals, and WBC: white blood cell.

## Data Availability

The data used to support the findings are included within the article.

## Supplementary Materials

Supplementary Materials Table S1: the sex and age of study individuals. Figure S1: gating strategy.

## References

[1] Vial T., Marti G., Misse D., Pompon J. (2021). Lipid interactions between flaviviruses and mosquito vectors. *Frontiers in Physiology*.

[2] Brito Ferreira M. L., Militao de Albuquerque M. F. P., de Brito C. A. A. (2020). Neurological disease in adults with zika and chikungunya virus infection in Northeast Brazil: a prospective observational study. *The Lancet Neurology*.

[3] Masmejan S., Musso D., Vouga M. (2020). Zika virus. *Pathogens*.

[4] Pielnaa P., Al-Saadawe M., Saro A. (2020). Zika virus-spread, epidemiology, genome, transmission cycle, clinical manifestation, associated challenges, vaccine and antiviral drug development. *Virology*.

[5] Abbink P., Stephenson K. E., Barouch D. H. (2018). Zika virus vaccines. *Nature Reviews Microbiology*.

[6] Fischer M., Lindsey N., Staples J. E., Hills S., Centers for Disease Control and Prevention CDC Prevention (2010). Japanese encephalitis vaccines: recommendations of the advisory committee on immunization practices (ACIP). *Morbidity and Mortality Weekly Report Recommendations and Reports*.

[7] Duehr J., Lee S., Singh G. (2018). Tick-borne encephalitis virus vaccine-induced human antibodies mediate negligible enhancement of Zika virus infection *in vitro* and in a mouse model. *mSphere*.

[8] Chang H. H., Huber R. G., Bond P. J. (2017). Systematic analysis of protein identity between Zika virus and other arthropod-borne viruses. *Bulletin of the World Health Organization*.

[9] Wang R., Wang X., Zhang L. (2022). The epidemiology and disease burden of children hospitalized for viral infections within the family Flaviviridae in China: a national cross-sectional study. *PLoS Neglected Tropical Diseases*.

[10] Vannice K. S., Hills S. L., Schwartz L. M. (2021). The future of Japanese encephalitis vaccination: expert recommendations for achieving and maintaining optimal JE control. *NPJ Vaccines*.

[11] Rathore A. P. S., St John A. L. (2020). Cross-reactive immunity among Flaviviruses. *Frontiers in Immunology*.

[12] Saron W. A. A., Rathore A. P. S., Ting L. (2018). Flavivirus serocomplex cross-reactive immunity is protective by activating heterologous memory CD4 T cells. *Science Advances*.

[13] Wen J., Wang Y. T., Valentine K. M. (2020). CD4^+^ T cells cross-reactive with dengue and Zika viruses protect against Zika virus infection. *Cell Reports*.

[14] Subramaniam K. S., Lant S., Goodwin L., Grifoni A., Weiskopf D., Turtle L. (2020). Two is better than one: evidence for T-cellcross-protection between dengue and Zika and implications on vaccine design. *Frontiers in Immunology*.

[15] Wang R., Zhen Z., Turtle L. (2020). T cell immunity rather than antibody mediates cross-protection against Zika virus infection conferred by a live attenuated Japanese encephalitis SA14-14-2 vaccine. *Applied Microbiology and Biotechnology*.

[16] Rivino L., Lim M. Q. (2017). CD4^+^ and CD8^+^T-cell immunity to dengue—lessons for the study of Zika virus. *Immunology*.

[17] Wen J., Elong Ngono A., Regla-Nava J. A. (2017). Dengue virus-reactive CD8^+^ T cells mediate cross-protection against subsequent Zika virus challenge. *Nature Communications*.

[18] Xia H., Wang Y., Atoni E., Zhang B., Yuan Z. (2018). Mosquito-associated viruses in China. *Virologica Sinica*.

[19] Zheng X., Yu X., Wang Y. (2020). Complete protection for mice conferred by a DNA vaccine based on the Japanese encephalitis virus P3 strain used to prepare the inactivated vaccine in China. *Virology Journal*.

[20] Zheng X., Yu X., Wang Y., Cui M., Wang R., Yin C. (2020). Immune responses and protective effects against Japanese encephalitis induced by a DNA vaccine encoding the prM/E proteins of the attenuated SA14-14-2 strain. *Infection, Genetics and Evolution*.

[21] Chen D., Duan Z., Zhou W. (2020). Japanese encephalitis virus-primed CD8^+^ T cells prevent antibody-dependent enhancement of Zika virus pathogenesis. *Journal of Experimental Medicine*.

[22] Huang H., Li S., Zhang Y. (2017). CD8^+^ T cell immune response in immunocompetent mice during Zika virus infection. *Journal of Virology*.

[23] Lima N. S., Moon D., Darko S. (2021). Pre-existing immunity to Japanese encephalitis virus alters CD4 T cell responses to Zika virus inactivated vaccine. *Frontiers in Immunology*.

[24] Erra E. O., Kantele A. (2015). The Vero cell-derived, inactivated, SA14-14-2 strain-based vaccine (Ixiaro) for prevention of Japanese encephalitis. *Expert Review of Vaccines*.

[25] Chin R., Torresi J. (2013). Japanese B encephalitis: an overview of the disease and use of Chimerivax-JE as a preventative vaccine. *Infectious Disease and Therapy*.

[26] Zhou C. M., Liu J. W., Qi R. (2020). Emergence of Zika virus infection in China. *PLoS Neglected Tropical Diseases*.

[27] Sallusto F., Geginat J., Lanzavecchia A. (2004). Central memory and effector memory T cell subsets: function, generation, and maintenance. *Annual Review of Immunology*.

[28] Zhang L., Liu M., Zhang M. (2022). Separation of immune cell subpopulations in peripheral blood samples from children with infectious mononucleosis. *Journal of Visualized Experiments*.

[29] Zhang L., Zhang M., Liu M. (2022). Detection of polyfunctional T cells in children vaccinated with Japanese encephalitis vaccine via the flow cytometry technique. *Journal of Visualized Experiments*.

[30] Wang R., Xie L., Gao N. (2019). Decreases in both the seroprevalence of serum antibodies and seroprotection against Japanese encephalitis virus among vaccinated children. *Virologica Sinica*.

[31] Hombach J., Solomon T., Kurane I., Jacobson J., Wood D. (2005). Report on a WHO consultation on immunological endpoints for evaluation of new Japanese encephalitis vaccines, WHO, Geneva, 2-3 September, 2004. *Vaccine*.

[32] Wang R., Liao X., Fan D. (2018). Maternal immunization with a DNA vaccine candidate elicits specific passive protection against post-natal Zika virus infection in immunocompetent BALB/c mice. *Vaccine*.

[33] Peng Y., Mentzer A. J., Liu G. (2020). Broad and strong memory CD4^+^ and CD8^+^ T cells induced by SARS-CoV-2 in UK convalescent individuals following COVID-19. *Nature Immunology*.

[34] Li C. K. F., Wu H., Yan H. (2008). T cell responses to whole SARS coronavirus in humans. *Journal of Immunology*.

[35] Grifoni A., Sidney J., Vita R. (2021). SARS-CoV-2 human T cell epitopes: adaptive immune response against COVID-19. *Cell Host & Microbe*.

[36] Gagnon S. J., Ennis F. A., Rothman A. L. (1999). Bystander target cell lysis and cytokine production by dengue virus-specific human CD4(+) cytotoxic T-lymphocyte clones. *Journal of Virology*.

[37] Wang R., Gao N., Li Y. (2019). Cross-protection against four serotypes of dengue virus in mice conferred by a Zika DNA vaccine. *Frontiers in Cellular and Infection Microbiology*.

[38] Wen J., Shresta S. (2017). T cell immunity to Zika and Dengue viral infections. *Journal of Interferon and Cytokine Research*.

[39] Yauch L. E., Zellweger R. M., Kotturi M. F. (2009). A protective role for dengue virus-specific CD8^+^ T cells. *Journal of Immunology*.

[40] Zellweger R. M., Eddy W. E., Tang W. W., Miller R., Shresta S. (2014). CD8^+^ T cells prevent antigen-inducedantibody-dependent enhancement of dengue disease in mice. *The Journal of Immunology*.

[41] PLoS Medicine Editors (2013). Poor health in rich countries: a role for open access journals. *PLoS Medicine*.

[42] Yauch L. E., Prestwood T. R., May M. M. (2010). CD4^+^ T cells are not required for the induction of dengue virus-specific CD8^+^ T cell or antibody responses but contribute to protection after vaccination. *The Journal of Immunology*.

[43] Aberle J. H., Koblischke M., Stiasny K. (2018). CD4 T cell responses to Flaviviruses. *Journal of Clinical Virology*.

[44] Wiesel M., Oxenius A. (2012). From crucial to negligible: functional CD8^+^T-cell responses and their dependence on CD4^+^T-cell help. *European Journal of Immunology*.

[45] Sheng S. Y., Gu Y., Lu C. G., Zou J. Y., Hong H., Wang R. (2017). The distribution and function of human memory T cell subsets in lung cancer. *Immunologic Research*.

[46] Garris C. S., Blaho V. A., Hla T., Han M. H. (2014). Sphingosine-1-phosphate receptor 1 signalling in T cells: trafficking and beyond. *Immunology*.

[47] Gagnon S. J., Zeng W., Kurane I., Ennis F. A. (1996). Identification of two epitopes on the Dengue 4 virus capsid protein recognized by a serotype-specific and a panel of serotype-cross-reactive human CD4^+^ cytotoxic T-lymphocyte clones. *Journal of Virology*.

[48] Lim M. Q., Kumaran E. A. P., Tan H. C. (2018). Cross-reactivity and anti-viral function of dengue capsid and NS3-specific memory T cells toward Zika virus. *Frontiers in Immunology*.

[49] Satchidanandam V. (2020). Japanese encephalitis vaccines. *Current Treatment Options in Infectious Diseases*.

[50] Lumley S. F., McNaughton A. L., Klenerman P., Lythgoe K. A., Matthews P. C. (2018). Hepatitis B virus adaptation to the CD8^+^ T cell response: consequences for host and pathogen. *Frontiers in Immunology*.

