# Supplementary figures and images for: Japanese Encephalitis Vaccine Generates Cross-Reactive Memory T Cell Responses to Zika Virus in Humans
Source: J Trop Med. 2022 Nov 19;2022:8379286. doi: 10.1155/2022/8379286 (PMC9701130; doi:10.1155/2022/8379286)

Supplementary material

Fig. S1 Gating strategy

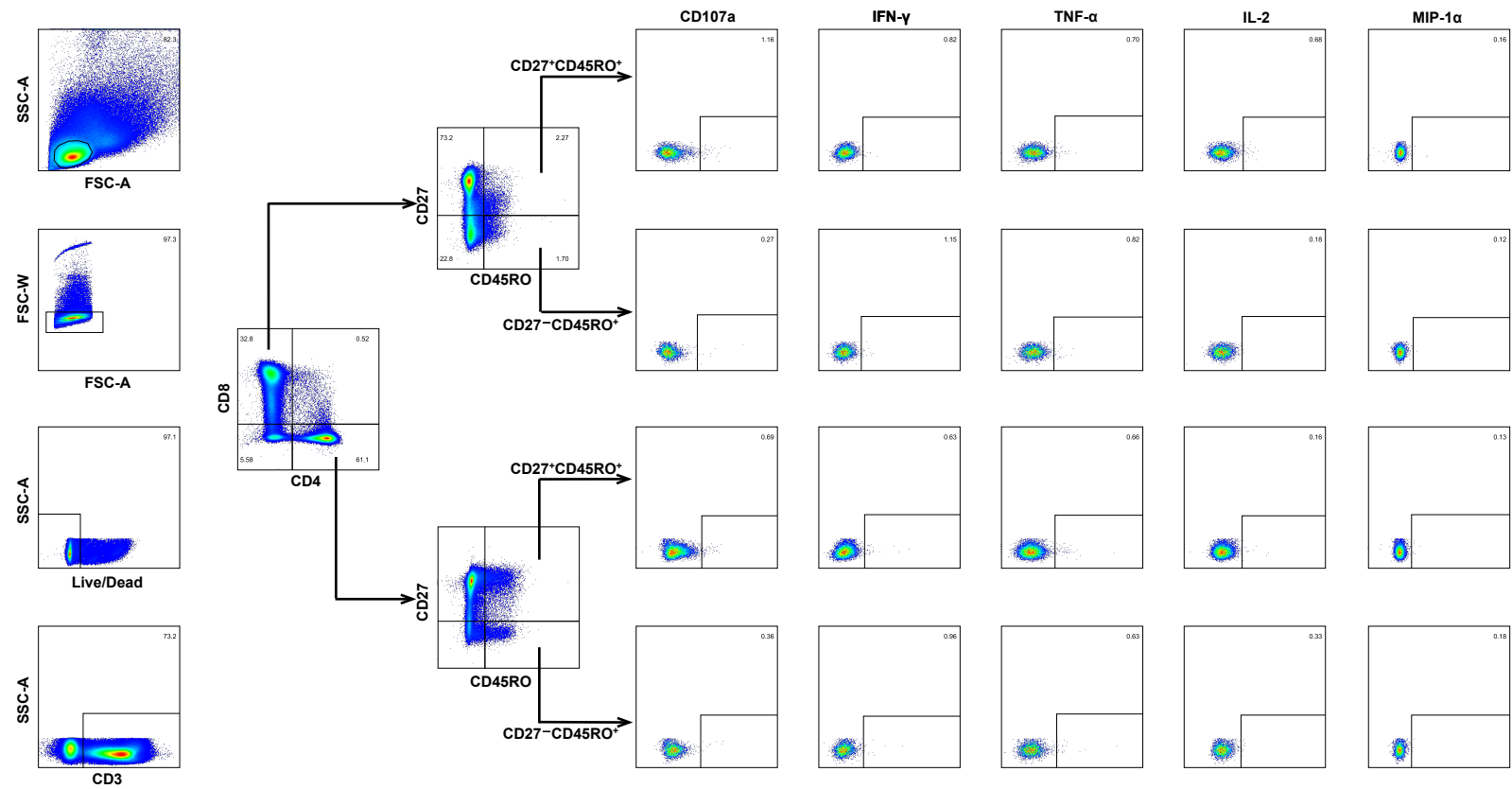

Supplement: Supplementary Materials — Table S1: the sex and age of study individuals. Figure S1: gating strategy. [file 8379286.f1.zip › Fig S1.pdf]
