# Supplementary material for: Japanese Encephalitis Vaccine Generates Cross-Reactive Memory T Cell Responses to Zika Virus in Humans
Source: J Trop Med. 2022 Nov 19;2022:8379286. doi: 10.1155/2022/8379286 (PMC9701130; doi:10.1155/2022/8379286)
Supplement: Supplementary Materials — Table S1: the sex and age of study individuals. Figure S1: gating strategy. [file 8379286.f1.zip › Table S1.doc]

**Table S1. Sex and age information of subjects in this study**

| **No.** | **Sex** | **Age** |
| --- | --- | --- |
| **VAC 1** | F | 2 y |
| **VAC 2** | F | 2 y |
| **VAC 3** | F | 2 y |
| **VAC 4** | M | 2 y |
| **VAC 5** | F | 2 y |
| **VAC 6** | M | 2 y |
| **VAC 7** | F | 2 y |
| **VAC 8** | F | 2 y |
| **VAC 9** | M | 2 y |
| **VAC 10** | F | 2 y |
| **VAC 11** | M | 2 y |
| **VAC 12** | F | 2 y |
| **VAC 13** | M | 2 y |
| **VAC 14** | M | 2 y |
| **VAC 15** | M | 2 y |
| **VAC 16** | F | 2 y |
| **VAC 17** | M | 2 y |
| **VAC 18** | M | 2 y |
| **UNV 1** | M | 6 m |
| **UNV 2** | F | 6 m |
| **UNV 3** | M | 6 m |
| **UNV 4** | M | 6 m |
| **UNV 5** | M | 6 m |
| **UNV 6** | F | 6 m |
| **UNV 7** | F | 6 m |

F: female

M: male

y: year

m: month
